# Human herpesvirus-7 meningitis in an immunocompetent adult patient: a case report

**DOI:** 10.2144/fsoa-2023-0021

**Published:** 2023-06-23

**Authors:** Rabih Fares, Madonna Matar

**Affiliations:** 1 Psychiatric Hospital of the Cross, Jal Eddib, 1525, Lebanon; 2 School of Medicine & Medical Sciences, Holy Spirit University of Kaslik, P.O. Box 446, Jounieh, Lebanon

**Keywords:** HHV-7, immunocompetent adult, meningitis

## Abstract

**Aim:**

The underlying pathological mechanisms of CNS human herpesvirus-7 (HHV-7) related infections are still unknown, especially among immunocompetent adults. Although HHV-7 meningitis in immunocompetent adults is usually uncommon, serious consideration for possible HHV-7 involvement should be taken when assessing CNS infection of unknown etiology.

**Case presentation:**

We report a 53-year-old female who presented for fever and progressive headaches. Cerebrospinal fluid (CSF) analysis was compatible with a viral meningitis. CSF cultures were negative and HHV-7 DNA was the only strain detected in the CSF analysis. The patient died 1 month later following complications and cardiac arrest.

**Conclusion:**

HHV-7 CNS infection in immunocompetent patient can be a serious infection. Prompt diagnosis and treatment management are essential for better outcome.

## Plain language summary

We do not fully understand how human herpesvirus-7 (HHV-7) brain infections affect healthy individuals. We share a case of a 53-year-old woman who presented with fever and worsening headaches. In her cerebrospinal fluid analysis, only HHV-7 DNA was found. She received treatment with acyclovir initially and later with ganciclovir for a total of 4 weeks. Unfortunately, the patient passed away 1 month later due to complications and cardiac arrest. We highlight the need for robust guidelines for effectively treating HHV-7 brain infections in healthy individuals.

## Tweetable abstract

Human herpesvirus-7 brain infection can lead to death in immunocompetent adults, a new report has found.

First reported in 1990, the human herpesvirus-7 (HHV-7) was classified in the subgroup of the Betaherpesvirinae, and is currently widespread, particularly among the pediatric population. In fact, 70% of children worldwide are infected with HHV-7 at some point before the age of 4 years via respiratory droplets or breast milk [1]. HHV-7 infections are usually asymptomatic or may cause *Exanthema subitum* [2]. The disease is usually self-limited, and no treatment is generally required [3]. However, serious neurological complications of HHV-7 infections, such as meningitis [4], encephalitis [5] and myelitis [6] can occur in rare cases, whether patients are immunosuppressed or immunocompetent, and little is known about the pathophysiology of CNS involvement in immunocompetent individuals [7]. There is no standard treatment protocol for neurological complications due to HHV-7 [8]. To our knowledge, this is the first reported case of death due to HHV-7 meningitis in an immunocompetent adult patient.

## Case report

A 53-year-old female patient was admitted to the emergency room with fever, progressive headaches, fatigue and constipation gradually developing since few days. She complained also of a recent urinary retention. Low-grade fever (38.3°C) started 2 days prior to presentation. She is known to have hypertension and diabetes mellitus treated with a synthetic oral angiotensin-II receptor antagonist and a combination of empagliflozin and metformin. The patient has taken three doses of Pfizer vaccine for COVID-19, the last one 5 months prior to her admission.

**Figure 1.**
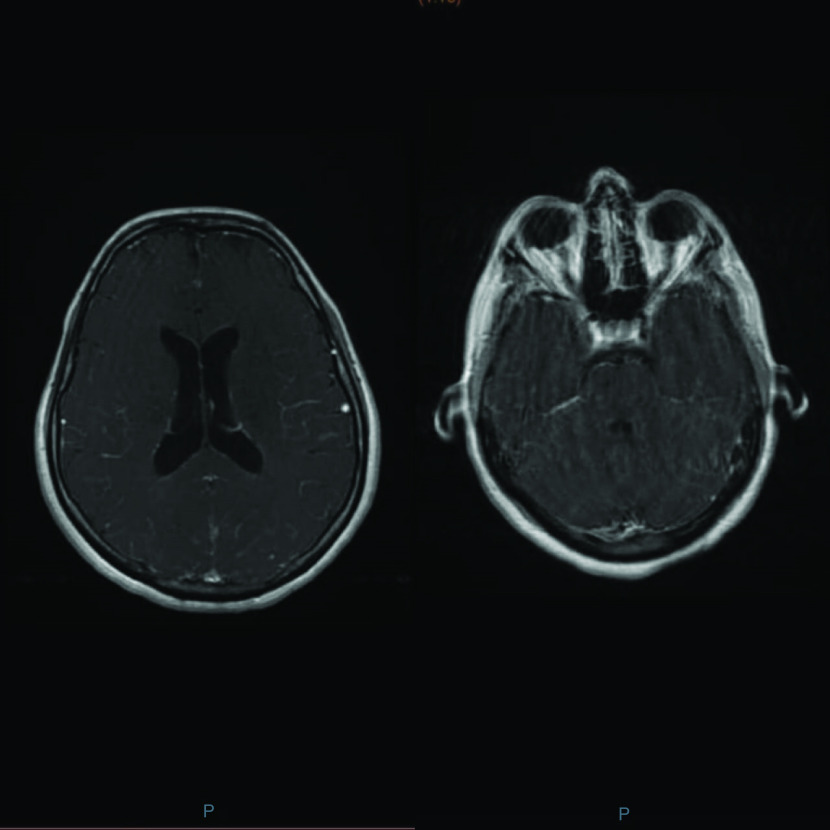
An MRI of the brain showing a diffuse leptomeningeal enhancement with hyperemia, compatible with the signs of meningitis.

On day 1, her temperature was 38.8°C, pulse 95 bpm, arterial blood pressure 100/50 mmHg, and a Glasgow Coma Scale (GCS) rate of 14/15, with confusion. On neurologic exam, the motor strength and control of both lower limbs was 4/5, with no other signs of neurological deficiency. The lungs were clear with no heart murmurs on cardiac exam. A light, diffuse tenderness of the abdomen was noted on exam, possibly related to her urinary retention. Her blood tests results showed normal white blood cells count of 5.810/mm^3^ with 62% of polymorphonuclear cells and 20% of lymphocytes and a normal C-reactive protein, creatinine and liver function tests. The chest x-ray result was normal. A multiplex PCR for COVID-19 was negative. An urgent MRI of the brain was requested in the emergency room, showing a diffuse leptomeningeal enhancement with hyperemia, compatible with the signs of meningitis (Figure 1). The lumbar puncture showed pleocytosis (900 cells/mm^3^) with 10% of polymorphonuclear cells and 90% of lymphocytes, glucose level of (41 mg/dl), protein level of (279.7 mg/dl) and albumin level (2095 mg/dl). Rapid antigen agglutination test for Hamophilus influenzae type B (HiB), Neisseria meningitidis, *Streptococcus pneumoniae* and *Escherichia coli* K1 were negative. Empirical therapy was started in the emergency room which included intravenous ceftriaxone iv. (2g Q12h), vancomycin (1g Q12h) and acyclovir iv. (10 mg/kg Q8h).

A PCR panel on CSF was requested in order to search for the possible etiologic agent. HSV 1–2, varicella zoster virus, EBV, CMV, human herpes virus-6, HHV-7, human adenovirus, human parechovirus, human enterovirus, mumps virus, parvovirus B19 (B19), neisseria meningitides, listeria monocytogenes, haemophilus influenzae, Streptococcus agalactiae (GBS), *S. pneumoniae* and *E. coli* K1 were requested. The test came back positive for HHV-7 on day 2, with a limit of detection of the assay about 100 copies/reaction.

On day 3, a HIV serology test was requested which returned negative and the antibiotics were dropped after the release of the negative cultures and the PCR panel. On day 4 of treatment, the patient remained lethargic with intermittent episodes of fever. A second MRI of the brain was requested to assess CNS lesions, which were noted as stable. Ampicillin iv. (2g Q4h) was added to her current regimen, while waiting for the listeria CSF culture results which were reported few days later as negative. A second lumbar puncture on day 5 was requested. The lumbar puncture showed pleocytosis (250 cells/mm^3^) with 20% of polymorphonuclear cells and 80% of lymphocytes, glucose level of 56 mg/dl, protein level of 85 mg/dl and albumin level of 529 mg/dl. The patient remained febrile (38.8°C) 1 week prior to her admission. Brucella serology and culture on CSF as well as tuberculosis DNA PCR were requested. They came back negative few days later. The clinical state of the patient began to deteriorate rapidly on day 8. She presented a GCS of 7 that required a rapid endotracheal intubation and transfer to the intensive care unit. The acyclovir iv. was discontinued, and ganciclovir iv. (5 mg/kg Q12h) was initiated. Meropenem (1g Q8h) was empirically added to cover possible sepsis related to resistant organisms. After multidisciplinary discussion, dexamethasone iv. was also initiated at a dose of 8 mg Q8h which was tapered every 48h to 6 mg Q8h, then 4 mg Q12h then stopped as no clinical amelioration was noted. Moreover, on day 9, and considering that the patient came from an endemic area for tuberculosis with a low CSF glucose level, a tuberculosis PCR on CSF was repeated and antituberculosis drugs were initiated empirically, consisting of isoniazid, rifampicin, pyrazinamide and ethambutol. The tuberculosis PCR was again negative and the tuberculosis culture came back negative 8 weeks later. On day 13, the GCS of the patient was still dropping, reaching a score of 4 and an electroencephalography was requested. It showed a persistent slow waves pattern. The patient died following cardiac arrest 15 days after her admission to the intensive care unit.

## Discussion

This is the first reported case of death of meningitis due to HHV-7 in an immunocompetent patient. Our patient presented with severe headaches, somnolence and confusion. At first, the differential diagnosis considered was meningoencephalitis. Brain MRI and lumbar puncture performed in the emergency room confirmed our suspected diagnosis. A review of diagnostic methods and guidelines for management of viral meningoencephalitis recommend that diagnosis of encephalitis should rely on epidemiologic, clinical and laboratory data, EEG and imaging [9].

Viral meningoencephalitis due to HHV-7 is frequently described among immunocompetent pediatric population and occasionally in immunocompromised adults. About 75% of children worldwide are HHV-7 seropositive before the age of 5 years [10] and the primo-infections with HHV-7 are still not very well understood [11]. However, the infection is not always benign in children and rare cases of sudden child death due to HHV-7 encephalitis were described [12]. Other neurological manifestations including Guillain–Barre syndrome [4], hemiplegia [13] and seronegative autoimmune encephalitis [14] were also described in immunocompetent children.

The same situation is not frequently found among immunocompetent adults, and neurological involvement due to HHV-7 is rarely reported. However, reactivations of HHV-7 infections among immunocompromised adults occur more frequently in some opportunistic infections and tumors. Sometimes, hyperglycemic state in some uncontrolled patients with diabetes may weaken their immune response and predispose them to reactivation of HHV-7 infections [15]. The underlying mechanism of reactivation of HHV-7 in immunocompromised adults is still unclear, and clinical outcomes of HHV-7 reactivation is still controversial, since HHV-7 reactivations in HIV positive individuals may lead to a better prognosis for unknown reason [16].

One recent retrospective study over a 4-year period, showed that among 251 immunocompetent adults with neurological symptoms, a total of six cases were related to HHV-7: four meningitis cases, one myelitis case and one encephalitis case [17]. Another two cases of meningoencephalitis due to HHV-7 were reported in Chile [18], two cases of encephalitis [5] in Italy with one of them involving the nucleus of the VI cranial nerve [19] and one case of limbic encephalitis in Japan [8]. Generally, the diagnosis was made by PCRs on body fluids. Such method detects the nucleic acid in CSF particularly when obtained during the first week of symptoms onset, with a specificity of 99% and a sensitivity of 96%, while these percentages drop significantly after the second week [20]. Taking into consideration that HHV-7 was detected in normal brains [18], some studies support the fact that HHV-7 was detected in pia mater meninges, frontal lobe, temporal lobe and olfactory tract of individuals with encephalopathy [21] and controversial studies are still debating the role of HHV-7 in the pathogenesis of the Alzheimer disease [22]. More research is required to understand the role of HHV-7 in determining prognosis in immunocompetent adults.

Regarding the treatment, the replication of HHV-7 *in vitro* was inhibited by foscarnet and ganciclovir. These two drugs were preferred when treating CNS infections due to HHV-7 [5,13]. However, another study reported that acyclovir could be beneficial in the treatment of HHV-7 meningoencephalitis [23]. In fact, it is common to see ganciclovir, acyclovir and foscarnet used in HHV-7 encephalitis [23]. One retrospective study showed that among 251 subjects, 14 had neurological manifestations due to HHV-7. Only foscarnet was found to be effective in the clearance of HHV-7 in CSF fluid, while ganciclovir was ineffective in the case of encephalitis [24]. Unfortunately, due to treatment limitation in Lebanon, foscarnet was not available in the market and therefore, was not used in the treatment of our patient. The only available antivirals in the market were acyclovir and ganciclovir. However, there is insufficient clinical report that support the fact that all these drugs are effective *in vivo* [25] and HHV-7 can be sometimes resistant to ganciclovir [26].

**Figure 2.**
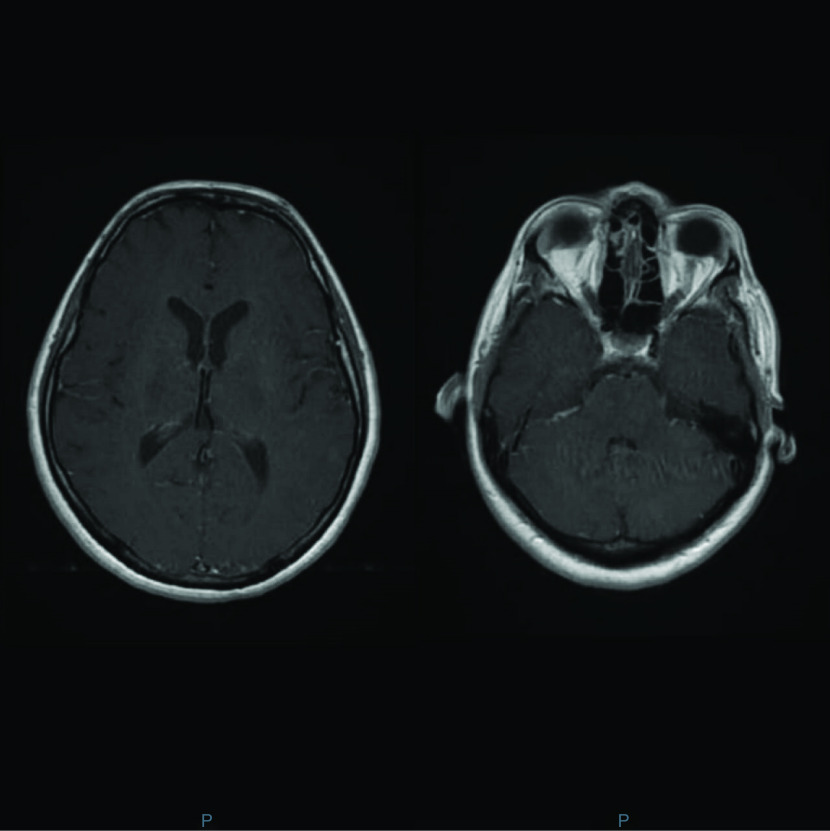
Similar imaging findings on brain MRI were noted 4 days later.

Our patient seemed to respond to treatment within the first 3 days before suddenly deteriorating. Treatment with acyclovir, followed by ganciclovir was continued for a total of 4 weeks. Noninfectious CNS diseases were excluded at her presentation. The finding of HHV-7 DNA in her CSF as well as the lymphocytes predominance in the CSF repeated twice, support the fact that HHV-7 was the principal etiologic cause of her meningitis. CSF pleocytosis was present and MRI of the brain showed a diffuse leptomeningeal enhancement with hyperemia, compatible with the signs of a meningitis. However, clinical amelioration of the patient was not observed neither with acyclovir, which was started empirically before the HHV-7 DNA PCR detection on CSF, nor with ganciclovir (Figure 2).

## Conclusion

To our knowledge, this is the first reported death case of meningitis due to HHV-7 in an immunocompetent adult. This case supports the neurotropic role of HHV-7 and CSF microbiological analysis should always include HHV-7 testing in patients developing signs of CNS involvement even if they are immunocompetent, for a better understanding of HHV-7 in the genesis of CNS pathology. The best treatment of HHV-7 meningitis remains unclear and future research are still needed for a better outcome of patients with meningitis due to HHV-7.

**Summary points**

- Human herpesvirus-7 (HHV 7) was classified in the subgroup of the *Betaherpesvirinae*, and is currently widespread, particularly among pediatric population.
- The disease is usually self-limited, and no treatment is generally required.
- Serious neurological complications of HHV-7 infections, such as meningitis, encephalitis and myelitis can occur in rare cases, whether patients are immunosuppressed or immunocompetent.
- There is no standard treatment protocol for neurological complications due to HHV-7.
